# Microglial Potassium Channels: From Homeostasis to Neurodegeneration

**DOI:** 10.3390/biom11121774

**Published:** 2021-11-26

**Authors:** Germana Cocozza, Stefano Garofalo, Riccardo Capitani, Giuseppina D’Alessandro, Cristina Limatola

**Affiliations:** 1 Instituto di Ricovero e Cura a Carattere Scientifico (IRCCS) Neuromed, 86077 Pozzilli, Italy; germana.cocozza@uniroma1.it (G.C.); giuseppina.dalessandro@uniroma1.it (G.D.); 2 Department of Physiology and Pharmacology, Sapienza University of Rome, 00185 Rome, Italy; stefano.garofalo@uniroma1.it (S.G.); riccardo.capitani@uniroma1.it (R.C.); 3 Department of Physiology and Pharmacology, Laboratory Affiliated to Istituto Pasteur Italia, Sapienza University of Rome, 00185 Rome, Italy

**Keywords:** microglia, potassium channel, CNS, neuroinflammation, neurodegenerative disease

## Abstract

The growing interest in the role of microglia in the progression of many neurodegenerative diseases is developing in an ever-expedited manner, in part thanks to emergent new tools for studying the morphological and functional features of the CNS. The discovery of specific biomarkers of the microglia phenotype could find application in a wide range of human diseases, and creates opportunities for the discovery and development of tailored therapeutic interventions. Among these, recent studies highlight the pivotal role of the potassium channels in regulating microglial functions in physiological and pathological conditions such as Alzheimer’s Disease, Parkinson’s Disease, and Amyotrophic Lateral Sclerosis. In this review, we summarize the current knowledge of the involvement of the microglial potassium channels in several neurodegenerative diseases and their role as modulators of microglial homeostasis and dysfunction in CNS disorders.

## 1. Introduction

Neurodegenerative diseases (NDDs) are disorders characterized by the progressive degeneration of the structure and function of the central or peripheral nervous systems. Recent research recognizes NDD, such as Alzheimer’s disease (AD), Huntington’s disease (HD), Parkinson’s disease (PD), frontotemporal dementia (FTD) and Amyotrophic Lateral Sclerosis (ALS) as the major causes of disability and the second leading cause of death worldwide [1]. Most NDDs are associated with a dysregulated immune response that impairs the CNS balance. Furthermore, neuroinflammation is an essential factor contributing to neurodegeneration, with reactive microglia playing a key role [2,3]. Microglia are the principal resident immune cells of the brain that support the physiological functions of the CNS. [4,5]. Furthermore, in the healthy CNS, microglia, which represent approximately 5–12% of CNS cells, are necessary for proper brain development, providing trophic support to neurons, removing apoptotic cell debris and regulating neuronal and synaptic plasticity [6,7,8,9]. Microglia are dynamic cells that shape their phenotype in a time- and context-dependent manner. In response to brain injury, microglia rapidly become reactive, assuming a detrimental or protective role. 

Microglial cells were historically described as assuming two opposite activation phenotypes, including the classical M1-like pro-inflammatorystate, and the alternative, M2-like, anti-inflammatory, state [10]. Now, evidence has shown that the M1/M2 model is inadequate to describe microglia activation in vivo [11]. Indeed, microglia show multiple phenotypes associated with aging, neurodegenerative diseases and disease stage. In pathological conditions microglia can (i) rapidly change their morphology and phenotype (ii) alter the transcriptional profile and the phagocytic activity; and (iii) modify their homeostatic functions [12,13,14,15,16]. In this way, microglia can play a detrimental role in the progression of several NDDs, expressing and secreting several receptors, cytokines and chemokines that recruit additional cells and clear toxic agents and cell debris, to maintain CNS homeostasis [17,18,19,20,21]. Highlighting the complexity of microglial functions in the scenario of NDDs, it was reported that microglial cells play different roles depending on the disease and on the stage of the pathology. Microglial cells mediate host defenses against danger signals, including pathogens and proteins, such as β-Amyloid (Aβ), mutant huntingtin, prions (PrPsc), and mutant or oxidized superoxide dismutase (SOD) [22,23,24]. At first, in response to pathological brain insults, microglia initiate a defense program to restore brain homeostasis via the expression and activities of microglial pattern recognition receptors (PRRs), including toll-like receptors (TLRs), scavenger receptors (SRs), and complement receptor 3 (CR3) [25,26,27,28,29,30,31,32]. Otherwise, when the perturbations persist, microglia can assume a detrimental phenotype, initiating an exaggerated inflammatory response, resulting in neurotoxicity and neurodegeneration. Accordingly, reactive detrimental microglia were found in the brain and spinal cord of NDDs patients at advanced stages of pathology [33,34]. For example, in ALS, activated microglia have been identified in close proximity to degenerating motor neurons, suggesting a direct neurotoxic function [35]. Using transcriptome profiling of disease-associated and homeostatic genes in microglia from the brain of the 5XFAD mouse model of AD, it was possible to identify a subpopulation defined as disease-associated microglia (DAM) [36]. DAM display downregulation of homeostatic microglial genes and the upregulation of genes involved in lysosomal, phagocytic, and lipid metabolism pathways, including several known disease risk factors, such as Apolipoprotein E (Apoe), Cystatin F(Ctsd 7), lipoprotein lipase (Lpl), Tyroprotein tyrosine kinase binding protein (Tyrobp), and triggering receptor expressed on myeloid cells 2 (Trem2) [36]. These DAMs are also found in other mouse models of neurodegeneration, including the tauopathy model Tau P301S, the SOD1^G93A^ model of ALS, the experimental autoimmune encephalomyelitis (EAE) model of multiple sclerosis (MS) and also during aging [37,38,39,40]. The heterogeneity and the complexity of microglia in normal and diseased CNS requires further studies to identify possible targets and strategies to modify microglial functions and restore their homeostasis in CNS disorders. Future advances about the roles of homeostatic microglia and DAM before and upon disease development will pave the way for new specific therapeutics. Since potassium channels expressed on microglial cells have been associated with a wide variety of neurodegenerative diseases, it is important to shed light on their regulation in neurodegeneration. 

## 2. Potassium Channels: Key Regulators of Microglial Function

Among the families of ion channels, the one of potassium (K^+^) channels represents the most abundant group, being involved in a multitude of physiological functions in both excitable and non-excitable cells [41]. Potassium channels have transmembrane helices (TMs) spanning the lipid bilayer [42], and regulate the flow of K^+^ ions across the cell membrane in different cells, such as neurons, glia, and lymphocytes [41].

### 2.1. Potassium Channels Expressed by Microglia

Many studies in vitro and in vivo/ex vivo demonstrated that human and rodent microglia express various types of K^+^ channels. Pioneer research on brain acute slice or primary cultures revealed that rodent microglia express voltage-gated (K_v_1.2, K_v_1.3, K_v_1.1, K_v_1.5, K_v_3.1), Ca^2+^-activated (K_Ca_3.1, K_Ca_2.3, K_Ca_1.1), and inward rectifying K^+^ channels (K_ir_2.1) [43]. More recently, the two-pore domain channel THIK-1 (K2P13.1) has been identified as a rodent microglia signature gene and is the main K+ channel in “resting” microglia [44]. On the other hand, it has been shown that Kv1.3, KCa3.1 and Kir2.1 are the main channels expressed by unstimulated mouse neonatal cultured microglia [45].

The expression of potassium channels in human samples has been mainly demonstrated by molecular and immunohistochemistry techniques in primary cultures and post-mortem brain tissues. Studies conducted on human fetal microglia cultures have shown the presence of a higher density of K^+^ currents with the biophysical and pharmacological components of K_v_1.3; while K_Ca_3.1 currents were found on microglia from adult human neocortical tissue [46,47,48,49]. K_v_1.3, K_Ca_3.1 and K_ir_2.1 expression has been reported on post-mortem human tissue [47].

In CNS, some microglial potassium channels are also expressed by other neuronal cell types. For instance, K_Ca_3.1 (*Kcnn4*) is expressed by transformed cells [50] and, in a model of spinal cord injury, it has been detected also on neurons and astrocytes [51]. Furthermore, K_v_1.3 (*Kcna3*) is found in addition to microglia, also in astrocytes, neurons of the olfactory system, and in gliomas [52,53,54,55].

### 2.2. Potassium Channels Modulation in Microglia

Potassium channel expression/activation can be regulated by many stimuli in vitro and in vivo, which make them promising targets in the treatment of several pathologies that their alteration correlates with.

Among pro-inflammatory stimuli the use of the TLR-4 ligand lipopolysaccharide (LPS) predominates in microglia studies. Hankyoung and colleagues reported for the first time that LPS treatment induced expression and function of K_v_1.5 and to a lesser extent Kv1.3 channels in rat primary cultures [56]. More recently, Nguyen et al. showed that pro-inflammatory or anti-inflammatory stimuli induced a different pattern of K+ channel expression in cultured neonatal mice and fetal human microglia [48]. The authors reported that neonatal mouse microglia stimulated with the LPS exhibited high K_V_1.3 current densities and virtually no K_Ca_3.1 and K_ir_ currents, while IL-4 stimulated microglia exhibited K_ir_2.1 currents and down-regulated K_V_1.3 and K_Ca_3.1 expression [48]. On the other hand, it has been reported that IL-4 up-regulated Kcnn4 mRNA expression and increased K_ca_3.1 current in rat and mouse neonatal cultured microglia [57,58]. However, Lam et al. have demonstrated the existence of many different responses of rat and mouse microglia to pro- and anti-inflammatory cytokines, also in terms of K channel expression [59]. Differences have been also reported comparing functional K channel expression in adult human and rodent microglia studies [49]. In vitro studies have reported an increased expression of K_v_1.1 and K_v_1.2 channels in microglia upon activation with ATP [60]. Other stimuli, such as gamma interferon (γ-IFN) or granulocyte macrophage colony stimulating (GMC-S) factor induced potassium currents in microglia cells, mostly those that are voltage-gate related. [61,62,63,64]. 

### 2.3. Cellular Functions Modulated by Microglial Potassium Channels

Upon their stimulation, microglial potassium channels regulate many cellular functions such as cell volume changes, membrane potential, hormone secretion, calcium signaling, gene expression and action potential firing [41,65]. By regulating membrane potential, cell volume and intracellular ion concentration, microglial K^+^ channels can affect proliferation, migration toward chemotactic stimuli, phagocytosis, morphology, and respiratory bursts [66]. Specifically, in microglia, K channel activation induces membrane hyperpolarization, which drives Ca^2+^ influx through inward rectifying Ca^2+^-Release-Activated-Ca^2+^ channels (CRAC) [45,67], ATP-activated P2X receptors [68] and other Ca^2+^-permeable cation channels [67]. Therefore, K^+^ channels facilitate the refilling of intracellular Ca^2+^ stores, thus maintaining a high Ca^2+^ content and the timing of intracellular signaling, and playing a crucial role in cell activation and proliferation [66,69]. Although this mechanism is presented as fully proved, very little is known about microglial Ca^2+^ signaling in situ or in vivo, both in the healthy and in the diseased brain.

The main reason is technical in nature, as microglia largely resisted in vivo labelling with small molecules to perform calcium imaging. However, today it is possible to study the link between potassium channel activation and calcium signaling directly thanks to new in vivo tools, such as genetically-encoded Ca^2+^ indicators (GECI) and high resolution two-photon microscopy [70,71,72]. Focusing on the functional expression of KCa3.1 in microglia, it has been reported that these channels mainly contribute to the Ca^2+^ activated K^+^ currents in the cell [73,74] and are involved in many cell functions. K_Ca_3.1 channels are involved in microglial migration, with cAMP/ PKA- and Ca^2+^ −dependent mechanisms [57,75], and in the production of reactive oxygen species (ROS) through the p38/MAPK and cGMP/PKG pathways [57,76]. Interestingly, K_Ca_3.1 and K_v_1.3 channels are involved in tumor/M2 polarized microglial cell activities, such as migration, phagocytosis, and gene expression modulation [77,78,79]. Concerning the K_v_1.3 channel, it has been demonstrated that this channel played a role in proliferation of freshly isolated hippocampal microglia [80]. Crucial microglia functions in brain homeostasis such as synaptic pruning, immunosurveillance and cytokine release are regulated by tonically active THIK-1 K^+^ channels expressed on cell plasma membranes. In particular, Izquierdo et al., demonstrated that THIK-1, using THIK-1–blocking drugs and THIK-1–deficient mice, regulates the removal of functional excitatory synapses by promoting microglial phagocytosis of synaptic material in the developing brain [81]. Considering the involvement of K^+^ channels in microglial function and morphology [66,82], we aim to summarize their contribution to the progress and development of neurological disorders, as follows.

## 3. Potassium Channels: Implications in NDDs

**Figure 1:**
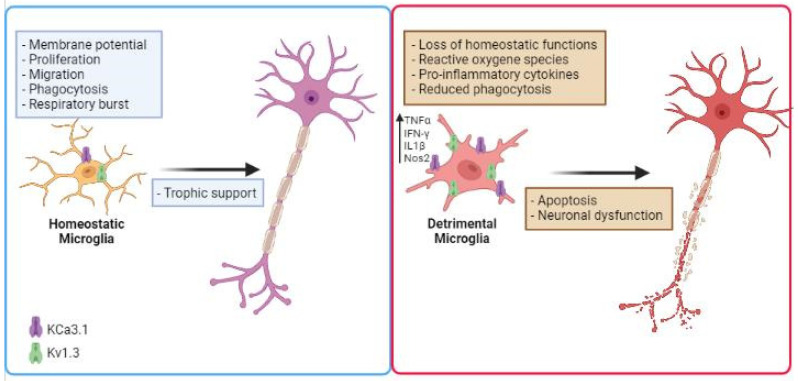
Schematic illustration describing the role of microglial K^+^ channels in NDD. Microglial cells increase the expression/activity of K_v_1.3 and K_Ca_3.1 in several neurodegenerative diseases, losing their homeostatic functions. This is associated with the release of inflammatory cytokines such as IFN-γ and TNF-α, production of noxious substances (ROS), reduced phagocytic activity, which are involved in inflammation-mediated neurotoxicity. Created in BioRender.com.

K^+^ channels have been involved in inflammation-mediated neurotoxicity [83]. The nature of the noxious stimuli in the CNS and their chronic production affects microglial functions, potentially leading to an exaggerated proinflammatory response, neurotoxicity, and neurodegeneration. In the last few years, several studies proposed to use K^+^ channel inhibitors to modulate microglial activation, to reduce inflammation in NDDs (Figure 1). Expression and functional alterations of K^+^ channels cause neuronal dysfunction and affect membrane excitability, contributing to the progress and development of many NDDs, such as AD, PD, ALS and other pathological conditions [84,85,86,87,88,89]. Two K^+^ channels, the calcium-activated K_Ca_3.1 and the voltage-gated K_v_1.3, play important roles in microglia activation by modulating Ca^2+^ signaling and membrane potential in CNS diseases. Because these channels are widely expressed and are implicated in immune cell activation, K_v_1.3 and K_Ca_3.1 inhibitors have been studied as potential targets for disease treatment. Before going on to review the role of microglia potassium channels in murine and human models of NDDs, it is important to mention that (i) several works observed critical differences between human and mouse microglia, especially in the context of aging and neurodegenerative conditions [90,91]; (ii) since the isolation of microglia presents several barriers, as microglia are highly sensitive to manipulations, slice cultures are recently used for human tissue to better preserve the environment. Furthermore, new techniques allow microglia phenotype characterization in live brain tissue as demonstrated in the work of Milior and colleagues [92].

*Alzheimer’s Disease:* Aberrant microglial activation represents a common pathological feature of several neurodegenerative diseases, including AD. Brain amyloid β deposition, a major pathological hallmark of the disease, can induce microglia activation. In this regard, an upregulation of K_v_1.3 and K_v_1.5 channels was found in Aβ-treated rat microglia and K_v_1.3 mediates Aβ-induced microglial ROS production and Aβ-induced microglial priming [93,94]. Of note, Maezawa et al, showed that pro-inflammatory and neurotoxic microglial responses induced by amyloid-β oligomer required K_v_1.3 activity in vitro and in hippocampal slices from mouse models of AD pathology [95]. Recent flow cytometric studies identified a distinct pro-inflammatory subset of CNS mononuclear phagocytes (CNS-MPs) that express the gene *Kcna3*, which encodes the K_v_1.3 K^+^ channel [96]. Interestingly, the expression of K_v_1.3 changes during disease, starting with a maximal increase at six months of age followed by a significant decrease between 10 and 12 months of age, a result that is accompanied by published electrophysiological data [95,96]. However, the downregulation of K_v_1.3 expression was not observed in human tissue: elevated K_v_1.3 channel expression has been observed in human postmortem AD brains by immunohistochemistry and by western blot analysis, and the elevation is limited to microglia, especially in those associated with amyloid plaques, suggesting that K_v_1.3 could still represent a relevant microglial target in AD [97]. Furthermore, long-term K_v_1.3 blockade in AD mouse models inhibits proinflammatory gene expression in microglia while promoting phagocytic uptake and clearance of Aβ [98]. It is possible that current transgenic models of disease perhaps do not completely replicate microglia phenotypes in human Alzheimer’s disease; further studies are needed to better define these discrepancies [97]. In addition to K_v_1.3, also K_Ca_3.1 was reported to play a role in AD. Recent studies demonstrated that the K_Ca_3.1 channel is upregulated in post-mortem sections of AD patients, and in 5XFAD transgenic AD mice starting from three to four months of age, at the insurgence of Aβ amyloidosis and microglial activation. [99]. In this work, the authors also showed that inhibition of K_Ca_3.1 mitigates some AD-like hallmarks including neuroinflammation, enhancing hippocampal neuronal plasticity and amyloid pathology. The same authors previously demonstrated that amyloid-β oligomers (ABO), at low (nanomolar) concentrations, increased microglia nitric oxide (NO) production, causing neuronal damage in vitro, in a K_Ca_3.1-dependent way [100].

*Parkinson’s Disease:* Many different studies demonstrated that neuroinflammation is critical to PD progression. The most important hallmark of PD is the presence of Lewy bodies, which contain aggregates of α-synuclein [101]. Microglial K_v_1.3 is transcriptionally upregulated in response to aggregated α-synuclein (αSynAgg) in primary microglial cultures and animal models of PD, as well as in postmortem human PD brains [102]. Recent evidence demonstrated that PD microglial K_Ca_3.1 also play a critical role in the progression of the disease. K_Ca_3.1 inhibition or gene deletion reduced dopaminergic (DA) neuron loss and improved the locomotor ability reducing microgliosis-mediated neuroinflammatory cytokine production in a mouse model of PD [103].

*Amyotrophic Lateral Sclerosis:* Several studies described that an inflammatory microenvironment contributes to motor neuron degeneration and disease progression in ALS [104,105], and we have recently shown that CNS infiltrating natural killer cells (NK) contribute to sustain an inflammatory microglia phenotype [21]. In accordance, we have described that in hSOD1^G93A^, a mouse model of familial ALS (fALS), spinal microglia overexpress K_Ca_3.1, and that the blockade of this channel induces a significant delay in the appearance of motor symptoms [89]. Specifically, a pharmacological blockade with K_Ca_3.1 inhibitor, 1- [(2-chlorophenyl)diphenylmethyl]-1H-pyrazole (TRAM-34), has beneficial effects in rodent models of ALS, reducing the expression of inflammatory factors such as TNF-α, Il-1β, Nos2 and increasing anti-inflammatory factors such as arg1, cd163, sosc3, ym1, bdnf and p2yr12 in the spinal cord. In line with the gene expression data, spinal microglia from TRAM-34-treated mice at the symptomatic stage are less amoeboid, have smaller soma and higher branching complexity, indicating that blockade of K_Ca_3.1 activity might restore the patrolling activity of microglia. Furthermore, TRAM-34 treatment delayed motor symptom appearance in the hSOD1^G93A^ ALS mouse model, as shown by prolonged muscle strength and motor coordination, and increased mice survival. Recently, we found that microglial K_Ca_3.1 is linked to hypothalamic neuroinflammation and affects feeding behaviour in ALS mouse models by restoring homeostatic microglia and attenuating weight loss [106]. It is important to note that a molecule structurally related to TRAM-34, Senicapoc, which has been previously found to be safe in humans in Phase I, II and III clinical trials [107,108] would be available for repurposing, and has been deposited by Pfizer for exactly this purpose in the National Center for Advancing Translational Research (NCATs) library.

### Other Pathological Conditions

*Ischemia:* Post-stroke inflammation plays an important role in brain tissue damage and microglia are the primary immune cells involved in this process, determining the severity of neuroinflammation. Microglia/macrophages acutely isolated from the infarct area in mice with middle cerebral artery occlusion (MCAO), a model for ischemic stroke, expressed Kir2.1, Kv1.3 and K_Ca_3.1 channels [47]. Furthermore, genetic deletion and pharmacological blockade of K_Ca_3.1, using TRAM-34, increased neuronal survival and reduced microglia activation and infarct size [47]. Subsequently, the same authors observed K_v_1.3 staining on activated microglia in ischemic infarcts in mice, rats, and humans, and found that K_v_1.3-inhibitor PAP-1 reduces secondary inflammatory damage after ischemia/reperfusion [109].

*Epilepsy* Several anti-inflammatory drugs have documented anticonvulsive effects, indicating the potential role of inflammation to the pathology of epilepsy [110]. Many studies report evidence that microglia are involved in epileptic processes. In particular, microglia seem to play a critical role, releasing increased levels of pro-inflammatory mediators, which may lead to neuronal hyperexcitability and neurodegeneration [110,111,112]. Studies on human microglia isolated from the temporal cerebral cortices of adult epileptic patients showed a higher expression of K_Ca_3.1 in comparison with non-epileptic brains [84,86]. Nevertheless, TRAM-34 treatment, in two different chronic epilepsy models, did not prevent seizures and even exacerbated pathology-related neuronal cell loss [113], thus suggesting that further studies are necessary to understand the effects of K_Ca_3.1 activity in epilepsy. Recently, it has been shown a predominant functional expression of K_v_1.3 channels in hippocampal microglia activated after status epilepticus in CX3CR1^eGFP/+^ mice [114].

*Cancer:* In Glioblastoma Multiforme (GBM), the most common primary brain tumor in the adults, microglia/macrophages (M/Mφ) represent the major infiltrating cell population. K_Ca_3.1 channels are attractive therapeutic targets for brain tumors, mainly because they are highly expressed in both GBM cells [50] and tumor associated microglia [58], while they are poorly expressed in normal CNS [115]. We have demonstrated that K_Ca_3.1 inhibition by shRNA or by pharmacological tools significantly reduced the tumor-infiltrated cerebral area, and also decreased astrogliosis and M/Mφ activation at the boundary of the tumor, suppressing M/Mφ phagocytosis and migration [116]. We also demonstrated that the K_Ca_3.1 channel blockade reduced tumor invasion and growth with direct effects on glioma, and it switches the activation state of tumor-associated microglia/macrophages (TAMs) towards an antitumor phenotype [78]. In addition, we reported that K_Ca_3.1 activation regulates microglia cell proliferation and neurotoxicity [58]. K_v_1.3 is involved in tumor associated astrocyte functions, indeed its inhibition reduced astrogliosis and increased glutamate clearance, reducing excitotoxic neuronal death. In addition, the selective inhibition of the channel reduced tumor growth and directly affected the invasive properties of tumor cells, confirming a key role of K_v_1.3 channels as modulators of glial cells [79].

## 4. Conclusions

In this review, we described the role of K^+^ channels in controlling microglia functions and maintaining brain homeostasis in health and disease. Neuroinflammation, driven by chronic glial cell activation, is often associated with a disturbance in the brain homeostasis that leads to NDD progression. The last few years have seen a significant progress in our understanding of the role of K^+^ channels in regulating microglia functions. Different microglial K^+^ channels have been associated with the onset and progression of various neurological disorders, and their physiological and pathological roles have been investigated. Microglia are often found near damaged tissue in NDDs patients (for example, microglia surrounding the Aβ plaques in AD or the degenerated motor neurons in ALS) and these channels on microglia have been linked to NDD onset and progression. Here we describe the knowledge on some of the most important potassium channels expressed by microglial cells and associated with neurological disorders, focusing on evidence on the potential beneficial effects of their blockade, deletion or silencing for therapeutic interventions. Taken together, such studies revealed that modulating microglia K^+^ channels can reduce microglia-mediated neuroinflammation, thereby preventing the apoptosis of neuronal cells, a mechanism that restrains immune microglia activation with consequent neuroprotective effects. New tools, especially single-cell RNA-sequencing and imaging technologies, will provide important new insights and will help to understand the physiological and pathological roles of microglial K+ channels both in animal models and human diseases. In conclusion, in this review we highlighted the evidence of the roles of K^+^ channels in regulating homeostatic microglia functions in several NDDs.

## References

[1] Feigin V.L., Nichols E., Alam T., Bannick M.S., Beghi E., Blake N., Culpepper W.J., Dorsey E.R., Elbaz A., Ellenbogen R.G. (2019). Global, regional, and national burden of neurological disorders, 1990–2016: A systematic analysis for the global burden of disease study 2016. Lancet Neurol..

[2] Perry V.H., Teeling J. (2013). Microglia and macrophages of the central nervous system: The contribution of microglia priming and systemic inflammation to chronic neurodegeneration. Sem. Immunopathol..

[3] Schwartz M., Kipnis J., Rivest S., Prat A. (2013). How do immune cells support and shape the brain in health, disease, and aging?. J. Neurosci..

[4] Nimmerjahn A., Kirchhoff F., Helmchen F. (2005). Resting microglial cells are highly dynamic surveillants of brain parenchyma in vivo. Science.

[5] Butovsky O., Jedrychowski M.P., Moore C.S., Cialic R., Lanser A.J., Gabriely G., Koeglsperger T., Dake B., Wu P.M., Doykan C.E. (2014). Identifcation of a unique TGF-β-dependent molecular and functional signature in microglia. Nat. Neurosci..

[6] Brown G.C., Neher J.J. (2014). Microglial phagocytosis of live neurons. Nat. Rev. Neurosci..

[7] Schafer D.P., Stevens B. (2013). Phagocytic glial cells: Sculpting synaptic circuits in the developing nervous system. Curr. Opin. Neurobiol..

[8] Tremblay M.E., Lowery R.L., Majewska A.K. (2010). Microglial interactions with synapses are modulated by visual experience. PLoS Biol..

[9] Paolicelli R.C., Bolasco G., Pagani F., Maggi L., Scianni M., Panzanelli P., Giustetto M., Ferreira T.A., Guiducci E., Dumas L. (2011). Synaptic pruning by microglia is necessary for normal brain development. Science.

[10] Hanisch U.K., Kettenmann H. (2007). Microglia: Active sensor and versatile effector cells in the normal and pathologic brain. Nat. Neurosci..

[11] Ransohoff R.M. (2016). A polarizing question: Do M1 and M2 microglia exist?. Nat. Neurosci..

[12] Chiu I.M., Morimoto E.T.A., Goodarzi H., Liao J.T., O’Keeffe S., Phatnani H.P., Muratet M., Carroll M.C., Levy S., Tavazoie S. (2013). A neurodegeneration-specifc gene-expression signature of acutely isolated microglia from an amyotrophic lateral sclerosis mouse model. Cell Rep..

[13] Wes P.D., Holtman I.R., Boddeke E.W., Moller T., Eggen B.J. (2016). Next generation transcriptomics and genomics elucidate biological complexity of microglia in health and disease. Glia.

[14] Plescher M., Seifert G., Hansen J.N., Bedner P., Steinhäuser C., Halle A. (2018). Plaque-dependent morphological and electrophysiological heterogeneity of microglia in an Alzheimer’s disease mouse model. Glia.

[15] Wake H., Moorhouse A.J., Jinno S., Kohsaka S., Nabekura J. (2009). Resting microglia directly monitor the functional state of synapses in vivo and determine the fate of ischemic terminals. J. Neurosci..

[16] Tam W.Y., Ma C.H. (2014). Bipolar/rod-shaped microglia are proliferating microglia with distinct M1/M2 phenotypes. Sci. Rep..

[17] Colonna M., Butovsky O. (2017). Microglia function in the central nervous system during health and neurodegeneration. Annu. Rev. Immunol..

[18] Kreutzberg G.W. (1996). Microglia: A sensor for pathological events in the CNS. Trends Neurosci..

[19] El Khoury J.B., Moore K.J., Means T.k., Leung J., Terada K., Toft M., Freeman M.W., Luster A.D. (2003). CD36 mediates the innate host response to beta-amyloid. J. Exp. Med..

[20] El Khoury J., Toft M., Hickman S.E., Means T.K., Terada K., Geula C., Luster A.D. (2007). Ccr2 defciency impairs microglial accumulation and accelerates progression of Alzheimer-like disease. Nat. Med..

[21] Garofalo S., Cocozza G., Porzia A., Inghilleri M., Raspa M., Scavizzi F., Aronica E., Bernardini G., Peng L., Ransohoff R.M. (2020). Natural killer cells modulate motor neuron-immune cell cross talk in models of Amyotrophic Lateral Sclerosis. Nat. Commun..

[22] Hickman S.E., Allison E.K., El Khoury J. (2008). Microglial dysfunction and defective beta-amyloid clearance pathways in aging Alzheimer’s disease mice. J. Neurosci..

[23] Liao B., Zhao W., Beers D.R., Henkel J.S., Appel S.H. (2012). Transformation from a neuroprotective to a neurotoxic microglial phenotype in a mouse model of ALS. Exp. Neurol..

[24] Muzio L., Martino G., Furlan R. (2007). Multifaceted aspects of inflammation in multiple sclerosis: The role of microglia. J. Neuroimmunol..

[25] El Khoury J., Hickman S.E., Thomas C.A., Cao L., Silverstein S.C., Loike J.D. (1996). Scavenger receptor-mediated adhesion of microglia to beta-amyloid fbrils. Nature.

[26] Hickman S.E., Kingery N.D., Ohsumi T.K., Borowsky M.L., Wang L., Means T.K., Khoury J. (2013). The microglial sensome revealed by direct RNA sequencing. Nat. Neurosci..

[27] Saijo K., Crotti A., Glass C.K. (2013). Regulation of microglia activation and deactivation by nuclear receptors. Glia.

[28] Heneka M.T., Golenbock D.T., Latz E. (2015). Innate immunity in Alzheimer’s disease. Nat. Immunol..

[29] Wu J., Chen Z.J. (2014). Innate immune sensing and signaling of cytosolic nucleic acids. Annu. Rev. Immunol..

[30] Areschoug T., Gordon S. (2009). Scavenger receptors: Role in innate immunity and microbial pathogenesis. Cell. Microbiol..

[31] Appel S.H., Zhao W., Beers D.R., Henkel J.S. (2011). The microglial-motoneuron dialogue in ALS. Acta Myol..

[32] Ransohof R.M., El Khoury J. (2015). Microglia in health and disease. Cold Spring Harb. Perspect. Biol..

[33] McGeer P.L., Itagaki S., Boyes B.E., McGeer E.G. (1988). Reactive microglia are positive for HLA-DR in the substantia nigra of Parkinson’s and Alzheimer’s disease brains. Neurology.

[34] Sapp E., Kegel K.B., Aronin N., Hashikawa T., Uchiyama Y., Tohyama K., Bhide P.G., Vonsattel J.P., DiFiglia M. (2001). Early and progressive accumulation of reactive microglia in the Huntington disease brain. J. Neuropathol. Exp. Neurol..

[35] Troost D., Van den Oord J.J., Vianney de Jong J.M. (1990). Immunohistochemical characterization of the inflammatory infiltrate in amyotrophic lateral sclerosis. Neuropathol. Appl. Neurobiol..

[36] Keren-Shaul H., Spinrad A., Weiner A., Matcovitch-Natan O., Dvir-Szternfeld R., Ulland T.K., David E., Baruch K., Lara-Astaiso D., Toth B. (2017). A unique microglia type associated with restricting development of Alzheimer’s disease. Cell.

[37] Lambert J.C., Ibrahim-Verbaas C.A., Harold D., Naj A.C., Sims R., Bellenguez C., DeStafano A.L., Bis J.C., Beecham G.W., Grenier-Boley B. (2013). Meta-analysis of 74,046 individuals identifies 11 new susceptibility loci for Alzheimer’s disease. Nat. Genet..

[38] Friedman B.A., Srinivasan K., Ayalon G., Meilandt W.J., Lin H., Huntley M.A., Cao Y., Lee S.-H., Haddick P.C.G., Ngu H. (2018). Diverse brain myeloid expression profiles reveal distinct microglial activation states and aspects of Alzheimer’s disease not evident in mouse models. Cell Rep..

[39] Krasemann S., Madore C., Cialic R., Baufeld C., Calcagno N., El Fatimy R., Beckers L., O’Loughlin E., Xu Y., Fanek Z. (2017). The TREM2-APOE pathway drives the transcriptional phenotype of dysfunctional microglia in neurodegenerative diseases. Immunity.

[40] Olah M., Patrick E., Villani A.C., Xu J., White C.C., Ryan K.J., Piehowski P., Kapasi A., Nejad P., Cimpean M. (2018). A transcriptomic atlas of aged human microglia. Nat. Commun..

[41] Miller C. (2000). An overview of the potassium channel family. Genome Biol..

[42] Kuang Q., Purhonen P., Hebert H. (2015). Structure of potassium channels. Cell Mol. Life Sci..

[43] Grissmer S., Nguyen A.N., Aiyar J., Hanson D.C., Mather R.J., A Gutman G., Karmilowicz M.J., Auperin D.D., Chandy K.G. (1994). Pharmacological characterization of five cloned voltage-gated K+ channels, types Kv1.1, 1.2, 1.3, 1.5, and 3.1, stably expressed in mammalian cell lines. Mol. Pharmacol..

[44] Madry C., Kyrargyri V., Arancibia-Cárcamo I.L., Jolivet R., Kohsaka S., Bryan R.M., Attwell D. (2018). Microglial ramification, surveillance, and interleukin-1β release are regulated by the two-pore domain K+ channel THIK-1. Neuron.

[45] Nguyen H.M., Blomster L.V., Christophersen P., Wulff H. (2017). Potassium channel expression and function in microglia: Plasticity and possible species variations. Channels.

[46] Eder C., Klee R., Heinemann U. (1997). Pharmacological properties of Ca2+−activated K+ currents of ramified murine brain macrophages. Naunyn-Schmiedeberg’s Arch. Pharmacol..

[47] Chen Y.J., Nguyen H.M., Maezawa I., Grössinger E.M., Garing A.L., Köhler R., Jin L., Wulff H. (2016). The potassium channel KCa3.1 constitutes a pharmacological target for neuroinflammation associated with ischemia/reperfusion stroke. J. Cereb. Blood Flow Metab..

[48] Nguyen H.M., Grössinger E.M., Horiuchi M., Davis K.W., Jin L.-W., Maezawa I., Wulff H. (2017). Differential Kv1.3, KCa3.1, and Kir2.1 expression in “classically” and “alternatively” activated microglia. Glia.

[49] Blomster L.V., Strøbaek D., Hougaard C., Klein J., Pinborg L.H., Mikkelsen J.D., Christophersen P. (2016). Quantification of the functional expression of the Ca(2+)-activated K+channel K_Ca_3.1 on microglia from adult human neocortical tissue. Glia.

[50] Kaushal V., Koeberle P.D., Wang Y., Schlichter L.C. (2007). The Ca2+-activated K+ channel KCNN4/KCa3.1 contributes to microglia activation and nitric oxide-dependent neurodegeneration. J. Neurosci..

[51] Bouhy D., Ghasemlou N., Lively S., Redensek A., Rathore K.I., Schlichter L.C., Samuel D. (2011). Inhibition of the Ca2+-dependent K+ channel, KCNN4/KCa3.1, improves tissue protection and locomotor recovery after spinal cord injury. J. Neurosci..

[52] Pannasch U., Färber K., Nolte C., Blonski M., Chiu S.Y., Messing A., Kettenmann H. (2006). The potassium channels Kv1.5 and Kv1.3 modulate distinct functions of microglia. Mol. Cell Neurosci..

[53] Nguyen T.D., Jeserich G. (1998). Molecular structure and expression of shaker type potassium channels in glial cells of trout CNS. J. Neurosci. Res..

[54] Fadool D.A., Tucker K., Perkins R., Fasciani G., Thompson R.N., Parsons A.D., Overton J.M., Koni P.A., Flavell R.A., Kaczmarek L.K. (2003). Expression of voltage-gated potassium channels Kv1.3 and Kv1.5 in human gliomas. Neurosci. Lett..

[55] Fadool D.A., Tucker K., Perkins R., Fasciani G., Thompson R.N., Parsons A.D., Overton J.M., Koni P.A., Flavell R.A., Kaczmarek L.K. (2004). Kv1.3 channel gene-targeted deletion produces “Super-Smeller Mice” with altered glomeruli, interacting scaffolding proteins, and biophysics. Neuron.

[56] Pyo H., Chung S., Jou I., Gwag B., Joe E.H. (1997). Expression and function of outward K+ channels induced by lipopolysaccharide in microglia. Mol. Cells.

[57] Ferreira R., Lively S., Schlichter L.C. (2014). IL-4 type 1 receptor signaling up-regulates KCNN4 expression, and increases the KCa3.1 current and its contribution to migration of alternative-activated microglia. Front. Cell. Neurosci..

[58] Grimaldi A., D’Alessandro G., Golia M.T., Grössinger E.M., Di Angelantonio S., Ragozzino D., Santoro A., Esposito V., Wulff H., Catalano M. (2016). KCa3.1 inhibition switches the phenotype of glioma-infiltrating microglia/macrophages. Cell Death Dis..

[59] Lam D., Lively S., Schlichter L.C. (2017). Responses of rat and mouse primary microglia to pro- and anti-inflammatory stimuli: Molecular profiles, K+ channels and migration. J. Neuroinflamm..

[60] Li F., Lu J., Wu C., Kaur C., Sivakumar V., Sun J., Li S., Ling E. (2008). Expression of Kv1.2 in microglia and its putative roles in modulating production of proinflammatory cytokines and reactive oxygen species. J. Neurochem..

[61] Norenberg W., Gebicke-Haerter P.J., Illes P. (1992). Inflammatory stimuli induce a new K+ outward current in cultured rat microglia. Neurosci. Lett..

[62] Norenberg W., Appel K., Bauer J., Gebicke-Haerter P.J., Illes P. (1993). Expression of an outwardly rectifying K+ channel in rat microglia cultivated on teflon. Neurosci. Lett..

[63] Langosch J.M., Gebicke-Haerter P.J., Norenberg W., Illes P. (1994). Characterization and transduction mechanisms of purinoceptors in activated rat microglia. Br. J. Pharmacol..

[64] Fischer H.G., Eder C., Hadding U., Heinemann U. (1995). Cytokine-dependent K+ channel profile of microglia at immunologically defined functional states. Neuroscience.

[65] Hille B. (2001). Ion Channels of Excitable Membranes.

[66] Kettenmann H., Hanisch U.-K., Noda M., Verkhratsky A. (2011). Physiology of Microglia. Physiol. Rev..

[67] Kraft R. (2015). STIM and ORAI proteins in the nervous system. Channels.

[68] Burnstock G. (2015). Physiopathological roles of P2X receptors in the central nervous system. Curr. Med. Chem..

[69] Michaelis M., Nieswandt B., Stegner D., Eilers J., Kraft R. (2015). STIM1, STIM2, and Orai1 regulate store-operated calcium entry and purinergic activation of microglia. Glia.

[70] Brawek B., Liang Y., Savitska D., Brawek B., Liang Y., Savitska D., Li K., Fomin-Thunemann N., Kovalchuk Y., Zirdum E. (2017). A new approach for ratiometric in vivo calcium imaging of microglia. Sci. Rep..

[71] Brawek B., Garaschuk O. (2013). Microglial calcium signaling in the adult, aged and diseased brain. Cell Calcium.

[72] Kato D., Ikegami A., Horiuchi H., Moorhouse A.J., Nabekura J., Wake H. (2019). In Vivo Two-Photon Imaging of Microglial Synapse Contacts. Methods Mol. Biol..

[73] Schilling T., Repp H., Richter H., Koschinski H.U., Dreyer F., Eder C. (2002). Lysophospholipids induce membrane hyperpolarization in microglia by activation of IKCa1 Ca(2+)- dependent K(+) channels. Neuroscience.

[74] Schilling T., Stock C., Schwab A., Eder C. (2004). Functional importance of Ca2+−activated K+ channels for lysophosphatidic acid induced microglial migration. Eur. J. Neurosci..

[75] Lively S., Schlichter L.C. (2013). The microglial activation state regulates migration and roles of matrix-dissolving enzymes for invasion. J. Neuroinflammat..

[76] Ferreira R., Wong R., Schlichter L.C. (2015). KCa3.1/IK1 channel regulation by cGMP-dependent protein kinase (PKG) via reactive oxygen species and CaMKII in microglia: An immune modulating feedback system?. Front. Immunol..

[77] Turner K.L., Honasoge A., Robert S.M., McFerrin M.M., Sontheimer H. (2014). A proinvasive role for the Ca(2+) -activated K(+) channel KCa3.1 in malignant glioma. Glia.

[78] D’Alessandro G., Grimaldi A., Chece G., Porzia A., Esposito V., Santoro A., Salvati M., Mainiero F., Ragozzino D., Di Angelantonio S. (2016). KCa3.1 channel inhibition sensitizes malignant gliomas to temozolomide treatment. Oncotarget.

[79] Grimaldi A., D’Alessandro G., Di Castro M.A., Lauro C., Singh V., Pagani F., Sforna L., Grassi F., D’Angelantonio S., Catacuzzeno L. (2018). Kv1.3 activity perturbs the homeostatic properties of astrocytes in glioma. Sci. Rep..

[80] Kotecha S.A., Schlichter L.C. (1999). A Kv1.5 to Kv1.3 Switch in Endogenous Hippocampal Microglia and a Role in Proliferation. J. Neurosci..

[81] Izquierdo P., Shiina H., Hirunpattarasilp C., Gillis G., Attwell D. (2021). Synapse development is regulated by microglial THIK-1 K+ channels. PNAS.

[82] Luo L., Song S., Ezenwukwa C.C., Jalali S., Sun B., Sun D. (2021). Ion channels and transporters in microglial function in physiology and brain diseases. Neurochem. Int..

[83] Fordyce C.B., Jagasia R., Zhu X., Schlichter L.C. (2005). Microglia Kv1.3 channels contribute to their ability to kill neurons. J. Neurosci..

[84] Palomba N.P., Martinello K., Cocozza G., Casciato S., Mascia A., Di Gennaro G., Morace R., Esposito V., Wulff H., Limatola C. (2021). ATP-evoked intracellular Ca 2+ transients shape the ionic permeability of human microglia from epileptic temporal cortex. J. Neuroinflamm..

[85] Villa C., Suphesiz H., Combi R., Akyuz E. (2019). Potassium channels in the neuronal homeostasis and neurodegenerative pathways underlying Alzheimer’s disease: An update. Mech. Ageing Dev..

[86] Villa C., Combi R. (2016). Potassium channels and human epileptic phenotypes: An updated overview. Front. Cell. Neurosci..

[87] Binda A., Rivolta I., Villa C., Chisci E., Beghi M., Cornaggia C.M., Giovannoni R., Combi R. (2018). A novel KCNJ2 mutation identified in an autistic proband affects the single channel properties of Kir2.1. Front. Cell. Neurosci..

[88] Thei L., Imm J., Kaisis E., Dallas M.L., Kerrigan T.L. (2018). Microglia in Alzheimer’s disease: A role of ion channels. Front. Neurosci..

[89] Cocozza G., Di Castro M.A., Carbonari L., Grimaldi A., Antonangeli F., Garofalo S., Porzia A., Madonna M., Mainiero F., Santoni A. (2018). Ca2+-activated K+ channels modulate microglia affecting motor neuron survival in hSOD1G93A mice. Brain Behav. Immun..

[90] Boche D., Perry V.H., Nicoll J.A.R. (2013). Activation patterns of microglia and their identification in the human brain. Neuropathol. Appl. Neurobiol..

[91] Galatro T.F., Holtman I.R., Lerario A.M., Vainchtein I.D., Brouwer N. (2017). Transcriptomic analysis of purified human cortical microglia reveals age-associated changes. Nat. Neurosci..

[92] Milior G., Chali F., Dos Santos T., Royer J., Miles R., Morin-Brureau M. (2019). Transcriptomics and Live Imaging to Define Functional Phenotypes of Microglia in Pathological Human Tissue. Methods Mol. Biol..

[93] Chung S., Lee J., Joe E.H., Uhm D.J. (2001). Beta-amyloid peptide induces the expression of voltage dependent outward rectifying K+ channels in rat microglia. Neurosci. Lett..

[94] Schilling T., Eder C. (2011). Amyloid-beta-induced reactive oxygen species production and priming are differentially regulated by ion channels in microglia. J. Cell Physiol..

[95] Maezawa I., Nguyen H.M., Di Lucente J., Maezawa I., Nguyen H.M., Lucente J., Jenkins D.P., Singh V., Hilt S., Kim K. (2018). Kv1.3 inhibition as a potential microglia-targeted therapy for Alzheimer’s disease: Preclinical proof of concept. Brain.

[96] Rangaraju S., Rangaraju S., Dammer E.B., Raza S.A., Rathakrishnan P., Xiao H., Gao T., Duong D.M., Pennington M.W., Lah J.J. (2018). Identification and therapeutic modulation of a pro-inflammatory subset of disease-associated-microglia in Alzheimer’s disease. Mol. Neurodegener..

[97] Rangaraju S., Gearing M., Jin L.W., Levey A. (2015). Potassium channel Kv1.3 is highly expressed by microglia in human Alzheimer’s disease. J. Alzheimers Dis..

[98] Ramesha S., Rayaprolu S., Bowen C.A., Giver C.R., Bitarafan S., Nguyen H.M., Gao T., Chen M.J., Nwabueze N., Dammer E.B. (2021). Unique molecular characteristics and microglial origin of Kv1.3 channel-positive brain myeloid cells in Alzheimer’s disease. Proc. Natl. Acad. Sci. USA.

[99] Jin L., Di Lucente J., Nguyen H.M., Singh V., Singh L., Chavez M., Bushong T., Wulff H., Maezawa I. (2019). Repurposing the KCa3.1 inhibitor senicapoc for Alzheimer’s disease. Ann. Clin. Transl. Neurol..

[100] Maezawa I., Zimin P.I., Wulff H., Jin L.-W. (2011). Amyloid-β protein oligomer at low nanomolar concentrations activates microglia and induces microglial neurotoxicity. J. Biol. Chem..

[101] Braak H., Del Tredici K., Rüb U., de Vos R.A.I., Steur E.N.H.J., Braak E. (2003). Staging of brain pathology related to sporadic Parkinson’s disease. Neurobiol. Aging.

[102] Sarkar S., Nguyen H.M., Malovic E., Langley M., Palanisamy B.N., Singh N., Manne S., Neal M., Gabrielle M., Abdalla A. (2020). Kv1.3 modulates neuroinflammation and neurodegeneration in Parkinson’s disease. J. Clin. Invest..

[103] Lu J., Dou F., Yu Z. (2019). The potassium channel KCa3.1 represents a valid pharmacological target for microgliosis-induced neuronal impairment in a mouse model of Parkinson’s disease. J. Neuroinflamm..

[104] Beers D.R. (2008). CD4+ T cells support glial neuroprotection, slow disease progression, and modify glial morphology in an animal model of inherited ALS. Proc. Natl. Acad. Sci. USA.

[105] Thonhoff J.R. (2018). Neuroinflammatory mechanisms in amyotrophic lateral sclerosis pathogenesis. Curr. Opin. Neurol..

[106] Cocozza G., Garofalo S., Morotti M., Chece G., Grimaldi A., Lecce M., Scavizzi, Menghini R., Casagrande V., Federici M. (2021). The feeding behaviour of ALS mouse models is modulated by the Ca2+ -activated KCa3.1 channels. Br. J. Pharmacol..

[107] Ataga K.I., Smith W.R., De Castro L.M., Swerdlow P., Saunthararajah Y., Castro O., Vichinsky E., Kutlar A., Orringer E.P., Rigdon G.C. (2008). Efficacy and safety of the Gardos channel blocker, senicapoc (ICA-17043), in patients with sickle cell anemia. Blood.

[108] Ataga K.I., Reid M., Ballas S.K., Yasin Z., Bigelow C., St James L., Smith W.R., Galacteros F., Kutlar A., Hull J.H. (2011). Improvements in haemolysis and indicators of erythrocyte survival do not correlate with acute vaso-occlusive crises in patients with sickle cell disease: A phase III randomized, placebo-controlled, double-blind study of the Gardos channel blocker senicapoc (ICA-17043). Br. J. Haematol..

[109] Chen Y.J., Nguyen H.M., Maezawa I., Jin L.W., Wulff H. (2018). Inhibition of the potassium channel Kv1.3 reduces infarction and inflammation in ischemic stroke. Ann. Clin. Transl. Neurol..

[110] Vezzani A., Lang B., Aronica E. (2016). Immunity and Inflammation in Epilepsy. Cold Spring Harb. Perspect. Med..

[111] Devinsky O., Vezzani A., Najjar S., De Lanerolle N.C., Rogawski M.A. (2013). Glia and epilepsy: Excitability and inflammation. Trends Neurosci..

[112] Hiragi T., Ikegaya Y., Koyama R. (2018). Microglia after Seizures and in Epilepsy. Cells.

[113] Ongerth T., Russmann V., Fischborn S., Katharina Boes K., Siegl C., Potschka H. (2014). Targeting of microglial K(ca)3.1 channels by TRAM-34 exacerbates hippocampal neurodegeneration and does not affect ictogenesis and epileptogenesis in chronic temporal lobe epilepsy models. Eur. J. Pharmacol..

[114] Menteyne A., Levavasseur F., Audinat E., Avignone E. (2009). Predominant functional expression of Kv1.3 by activated microglia of the hippocampus after Status epilepticus. PLoS ONE.

[115] Richardson P.J. (2016). CXCR4 and Glioblastoma. Anticancer. Agents Med. Chem..

[116] D’Alessandro G., Catalano M., Sciaccaluga M., Chece G., Cipriani R., Rosito M., Grimaldi A., Lauro C., Cantore G., Santoro A. (2013). KCa3.1 channels are involved in the infiltrative behavior of glioblastoma in vivo. Cell Death Dis..

